# Concussion history associated with adolescent psychological distress but not hazardous gambling: a cross-sectional study

**DOI:** 10.1186/s40359-024-01830-6

**Published:** 2024-06-05

**Authors:** Mitchell J. Andersson, Sabina Kapetanovic, Anders Håkansson, Emma Claesdotter-Knutsson

**Affiliations:** 1 Faculty of Medicine, Department of Clinical Sciences, Psychiatry, Lund University, Lund, Sweden; 2 Clinical Sports and Mental Health Unit, Malmö Addiction Center, Region Skåne, Malmö, Sweden; 3 Department of Social and Behavioral Sciences, University West, Trollhättan, Sweden; 4 Department of Psychology, Stockholm University, Stockholm, Sweden; 5 Child and Adolescent Psychiatry Outpatient Clinic, Region Skåne, Lund, Sweden

**Keywords:** Concussion, Traumatic brain injury, psychological distress, Gambling, Adolescence

## Abstract

**Background:**

Sustaining multiple concussions over one’s lifetime may be associated with behavioral and mood changes beyond the acute phase of injury. The present cross-sectional study examined the relationship between concussion history, the incidence of current moderate-severe psychological distress, and lifetime adolescent hazardous gambling in high school students.

**Methods:**

Four-hundred fifty-nine high school students from southern Sweden (age: 16.81 ± 0.83, 58.2% male) completed a survey assessing concussion history (0,1,2…>8), psychological distress using the Kessler-6 scale, and lifetime hazardous gambling using the NODS-CLiP scale.

**Results:**

Participants who self-reported three or more concussions were more likely to endorse moderate-severe symptoms of psychological distress than those with no concussion history while controlling for covariates, *OR* = 2.71, 95% CI [1.19, 6.18]. In contrast, concussion history was not associated with hazardous gambling after controlling for confounding variables.

**Conclusions:**

Self-reporting three or more concussions was associated with increased current psychological distress beyond the acute phase of injury among high school students. Adolescents who have sustained multiple concussions should undergo mental health evaluations beyond the acute phase of injury to identify and treat psychological distress, but probing for hazardous gambling may not be clinically relevant in this previously concussed adolescent population.

## Background

Up to 30 million brain injuries occur worldwide each year [1]. Concussion, often used interchangeably with mild traumatic brain injury (mTBI), is a brain injury caused by a direct or indirect blow to the head, neck, or body that transmits significant biomechanical force to the head and brain [2]. This force precipitates a cascade of neurobiological changes and subsequent neurologic dysfunction lasting for days to weeks following injury [3]. In contrast to our well-established understanding of concussion symptomology in the acute phase of injury (< 1 month), the long-term consequences of concussion on cognitive, physical, and psychological health are still the subject of intense debate and remain at the forefront of research [4]. Recent research, despite a lack of consensus, suggests a dose-dependent relationship between concussion, specifically sports-related concussion (SRC), and the later development of depression, emotional disturbance, sleep abnormalities, and addictive behaviors [5–13].

Depression and anxiety, sometimes grouped and defined as psychological distress, are among the most common mood-related symptoms reported by adolescents post-concussion [14]. These symptoms often spontaneously resolve within a month, but many patients continue to present with symptoms beyond the acute phase of injury [15, 16], or these symptoms slowly reemerge after a period of remission. A meta-analysis of 89,114 children with concussion by Gornall and colleagues [5] found that pediatric concussion was associated with increased odds of exhibiting internalizing symptoms, such as depression and anxiety beyond three months post-injury. Additionally, a recent retrospective cohort study conducted by Ledoux and colleagues [8] compared 152,321 children with concussion to 296,482 age and gender-matched controls with orthopedic injuries. Their study revealed that both single and multiple concussions were linked to a higher risk of receiving a mental health diagnosis over time compared to sustaining an orthopedic injury. Despite these large-scale studies, there is a dearth of high-quality studies demonstrating how this relationship is influenced by individual factors in, specifically, older adolescent student populations who are more likely to begin experiencing symptoms of depression and anxiety [17].

Research on behavioral addictions, specifically gambling for money, has expanded tremendously since the addition of non-substance-related disorders in the Diagnostic and Statistical Manual of Mental Disorders (DSM-5) [18]. Gambling disorder (GD), is an addictive disorder characterized by a “persistent and recurrent problematic gambling behavior leading to clinically significant impairment or distress” [18 pp. 585], which affects approximately 5.8% of adolescents and 7.0% of elite athletes in Sweden [19, 20]. In Sweden, gambling for money is forbidden for individuals under the age of 18, though many minors find avenues to engage in gambling activities [19]. Due to its prohibition for minors, prevalence metrics may underestimate the occurrence of adolescent gambling behavior and GD.

GD is characterized by tolerance, an inability to abstain from gambling activities, preoccupation with gambling, chasing losses, lying to conceal gambling activity, and reliance on others for financial support due to gambling [21]. In addition to significant psychosocial and financial distress, those with GD have a lower quality of life and a higher suicide risk [21]. The etiology and course of GD are complex, and researchers have only recently begun to uncover the role that brain injury and concussion play in the development of GD. As emphasized in Olsen and Corrigan’s examination of TBI as a precursor to substance use and substance use disorders [13], there exists a significant increase in risky substance use after the initial stage of TBI-injury. They posit that TBI fosters risky substance use through physiological mechanisms linked to persistent alterations in neuroplasticity, neuroimmune signaling, and network connectivity, all of which could potentially be generalized to non-substance use addictive disorders, such as GD. In fact, researchers have taken an interest in measuring how TBI may first engender hazardous gambling, a pattern of gambling that resembles and often precedes GD, albeit not at the severity required to satisfy criteria for a GD diagnosis [22]. Previous studies have demonstrated that moderate-to-severe TBI independently predicts subsequent hazardous gambling in Canadian adults and adolescents [23–26], as well as American veterans [27] which may be attributable to TBI-induced impaired risk-based decision-making and increased motor impulsivity as evidenced by preclinical trials using rodent models [28, 29]. For instance, Shaver and colleagues [29] conducted a preclinical experiment where they exposed 4-month-old rats to either a controlled cortical impact or a sham procedure. They then monitored the rats’ performance in a task resembling the Iowa Gambling Task over the course of 12 weeks. Their findings revealed that rats subjected to the cortical impact opted for suboptimal alternatives more often than sham controls, indicating impaired risk-based decision-making. Despite growing evidence linking more severe forms of TBI and later hazardous gambling in various age-groups and demographics, no study hitherto has assessed the relationship between concussion history and adolescent hazardous gambling.

Adolescence is a critical stage of physical and psychosocial development during which high-risk behaviors, like gambling, are popular and pervasive among certain subgroups [30–32]. Hazardous gambling during this stage may lead to a myriad of immediate consequences that hinder development and ultimately have a negative impact on the individual long-term. For example, adolescents who exhibit hazardous gambling are more likely to have poorer school performance and drop out, have familial issues at home, have weakened intimate relationships, engage in illicit substance use, and experience career disruptions that are at least partially attributable to the consequences of earlier adolescent hazardous gambling [33, 34]. Attenuating the burden of adolescent gambling on the individual and society is therefore of high priority.

### Aims and hypotheses

Based on previous research indicating that multiple concussions in an individual’s lifetime leads to poorer mental health outcomes [5–9], that adolescence is a transitional period when critical development and risky behavior (i.e., illicit gambling) occur [30–32], and that GD is associated with poorer quality of life and suicide [21], understanding the genesis of hazardous gambling and psychological distress in adolescent populations and its relationship to concussion is essential. Thus, the purpose of the present study is to assess the impact of concussion history on psychological distress and hazardous gambling behavior in youth populations while controlling for known covariates, namely age, sex, body mass index (BMI), athlete status, sport type, current and previous parental gambling, as well as previous diagnosis of attention deficit hyperactivity disorder (ADHD), autism spectrum disorder (ASD), and learning disorders (LD). The inclusion of these variables was based on existing literature indicating that older adolescents, males, those with higher BMI, athletes, those with parents who have a history of gambling, and those with psychiatric disorders are more likely to exhibit hazardous gambling behavior [35–38]. Several of these factors are also associated with increased odds of sustaining concussion and developing persisting concussive symptoms [5, 16, 39, 40]. We hypothesize that (1) students reporting more previous concussions will be more likely to report greater psychological distress and exhibit hazardous gambling behavior (2) that these relationships will remain significant when controlling for individual differences. Results will contribute to the literature mapping the potential post-acute effects of concussion on mental health and addictive behavior.

## Methods

### Participants

Each of the twenty high schools with at least one sports-tailored program located in Skåne county, Sweden were invited to participate. Eleven of twenty principals (55%) agreed to distribute the survey link to students through their respective instructors in class. A total of 661 participants consented and continued onto the survey, of which 463 (70%) provided a response to at least half of items. Four participants reported sustaining a concussion in the previous month and were excluded from further analysis to avoid assessing the consequences of acute concussion symptoms on outcome measures, leading to a final sample size of 459 participants.

### Procedure

The present observational study utilized a cross-sectional survey design. High school principals representing a school district in Skåne County, Sweden offering both traditional and sports-tailored curriculums were contacted via email and telephone. In Sweden, sports-tailored high school programs integrate the traditional high school curriculum with the training and competition schedules of student-athletes. Student-athletes can apply to tailored programs at the national, regional, and local levels, with the level of competition and demand on student-athletes depending on the program’s level [41]. All students attending these high schools were invited to participate, regardless of whether they were enrolled in tailored or traditional curricula or competed at the national, regional, or local levels. Students typically enter high school at the age of 16 and complete their studies within three years. Students were informed of the goals of the study and that their participation was voluntary and anonymous before entering the survey. Data collection took place between January 16th and February 17th, 2023. A reminder to participating principals was sent two weeks prior to closing the survey. Ethical approval for the present project was sought through the Swedish Ethical Review Authority, who came to the decision that formal ethical approval was not required because no personal identifiable data were to be collected (#DNr: 2022-04648-01, October 30th, 2022). In accordance with Swedish Law concerning the ethical review of research involving humans (SFS 2003:460 [42]), only students 15 years or older were invited to participate. Study procedures adhered to the Declaration of Helsinki and participants provided digital informed consent prior to proceeding to the survey.

### Measures

#### Psychological distress

Non-specific psychological distress was assessed using the Swedish-translated Kessler-6 scale (K6) [43], which is the short-form of the K10 scale whose Danish translation has been validated [44]. The K6 scale consists of six items pertaining to how often an individual has experienced depression- and anxiety-related symptoms over the past six months. Responses were given on a five-point Likert-type scale ranging from “not at all” (0) to “all the time” (4). Item scores were summed (0–24) and grouped based on Furukawa and colleagues’ [45] suggestions: no/low distress (0–7), moderate distress (8–12), and severe distress (13–24). Internal consistency of the scale in the present study was high (Cronbach’s α = 0.87).

#### Hazardous gambling

Lifetime hazardous gambling was assessed using the three-item version of the National Opinion Research Center DSM-IV Screen for Gambling Problems – Control, Lying, and Preoccupation (NODS-CLiP) [46, 47]. The screen includes three items probing for the incidence of loss of control, lying, and preoccupation associated with gambling during the participant’s lifetime, to which they respond on a binary “Yes” or “No” scale. Endorsing one of the three behaviors in relation to one’s gambling results in a positive screening for lifetime hazardous gambling. The NODS-CLiP has ample sensitivity and is preferable to other iterations of the original NODS scale, such as the NODS-PERC, in general population samples where the prevalence rate of hazardous gambling is low [47].

#### Individual differences

Demographics pertaining to age, sex, height, weight, athlete status, sport type, current and previous parental gambling, as well as previous ADHD, ASD, or LD diagnoses were collected to serve as covariates based on prior research [35–38, 48]. Height and weight were used to calculate participant BMI. Athlete status was coded on a binary scale based on student enrollment in sports-tailored academic programs. Reporting of at least one current or prior ADHD, ASD, or LD diagnosis was coded on a binary scale. Parental gambling behavior was assessed with two items, “Has your mother/father gambled in the last 12 months” (No, never; Yes, occasionally; Yes, often; Do not know). Responses were transformed to be binary. If participants reported that either of their parents gambled occasionally or gambled often, responses were coded as positive (1), while those that indicated that their parents did not gamble or did not know were coded as negative (0).

#### Concussion history

Participants were presented with the following commonly used definition of concussion to assess concussion history, “A concussion is an injury occurring typically, but not necessarily, from a blow to the head, followed by a variety of symptoms that may include any of the following: headache, dizziness, loss of balance, blurred vision, ‘seeing stars,’ feeling in a fog or slowed down, memory problems, poor concentration, nausea, throwing up, and loss of consciousness. Getting ‘knocked out’ or being unconscious does not always occur from a concussion” [49, pp.2]. Participants were asked based on this definition how many times they had sustained a concussion, both diagnosed and undiagnosed. Reponses were given on an interval scale (0, 1, 2… 8+), but were ultimately stratified into 0, 1–2, and 3+ concussions, as was predetermined based on contemporary concussion research [50, 51]. Participants were also asked when their latest concussion occurred to control for the acute effects of concussion injury on outcome measures.

### Statistical analyses

Missing data were processed using both simple and multiple imputation strategies. Missing data for K6 items were imputed with participants’ scale median, while missing demographic data were imputed via logistic regression for binary variables (e.g., sex) and linear regression for continuous variables (e.g., height and weight) through multiple imputation by chained equations with the *mice()* package. Assumptions of multicollinearity and linearity were assessed using VIF values and scatterplots of the log odds of the dependent variable against continuous predictor variables, respectively. BMI was not related to hazardous gambling nor psychological distress logits and was therefore not included in our logistic regression models. The assumption of no outliers was met as assessed by Cook’s distance. Age and BMI were not normally distributed, as assessed by Shapiro-Wilk’s tests (*p* < .05). Therefore, demographic variables were compared across concussion groups using the non-parametric alternative to analysis of variance, Kruskal-Wallis H test, for continuous variables and Chi-square (*χ*^*2*^) analyses for binary variables. The Benjamini-Hochberg procedure was carried out on *p*-values to account for increased risk of Type I error from multiple comparison across groups [52].

Thereafter, we conducted a series of binary logistic regressions predicting psychological distress with concussion history independently in Model 1a and alongside important covariates in Model 2a (age, BMI, sex, athlete status, and psychiatric history). We then conducted penalized logistic regressions with Firth’s bias reduction method [53] and *post hoc* intercept adjustments [54] to predict NODS-CLiP results with concussion history as an independent predictor in Model 1b as well as when controlling for important covariates in Model 2b (age, BMI, sex, athlete status, psychiatric history, parental gambling). A Firth regression was preferred due to the rareness of hazardous gambling in this population, which can lead to separation in logistic regression [55]. Rv4.2.1 was used to conduct all analyses, including the *logistf()* [56], *stats()*, and *car()* packages.

## Results

### Participants

Missing data for participants were minimal (2.2%, 613/27,317) and deemed missing at random based on visual inspection of missing data patterns. Participants were 16.81 ± 0.83 years old with a BMI of 22.08 ± 2.88 $\frac{kg}{{m}^{2}}$. Most were male (*n* = 267, 58.2%), in their first year of secondary school (*n* = 219, 47.8%), enrolled in a sports-tailored high school curriculum (*n* = 242, 52.7%), and lived at home with parents, step-parents, or caregivers (*n* = 437, 95.2%). Fifteen students reported an ADHD diagnosis, four students reported an ASD diagnosis, and 37 students reported having a LD (e.g., dyslexia). Approximately half (47.3%) of all students reported any form of gambling across their lifetime, while 24.6% reported gambling in the past 30 days. The most popular gambling types were in-person cards or poker (30.3%_*lifetime*_, 10.0%_*current*_), online sports betting (20.7%_*lifetime*_, 9.6%_*current*_), and within computer game gambling (i.e., to win real money or play the game; 20.0%_*lifetime*_, 7.2%_*current*_).

Overall, participant mean K6 scores were 7.52 ± 4.87 (0–24), with 113 (24.6%) participants endorsing moderate symptoms and 69 (15.0%) endorsing severe symptoms of psychological distress. On the other hand, 41 (8.9%) participants were identified as hazardous gamblers according to NODS-CLiP results, with 24 (5.2%) reporting loss of control over their gambling, 17 (3.7%) reporting lying to conceal their gambling, and 22 (4.8%) reporting preoccupation of gambling-related thoughts. A similar percentage of students enrolled in sports-tailored programs and traditional curricula were identified as hazardous gamblers (8.3%_*sports−tailored*_ vs. 9.7%_*traditional*_). Of participants enrolled in a sports-tailored high school curriculum, there was no difference in the prevalence of hazardous gambling between team sport athletes (*n* = 14, 8.0%) and individual sport athletes (*n* = 6, 9.0%), χ^2^(1) = 0.058, *p* = .81, *p*_*adj*_ = 0.99.

Most participants reported no previous concussions (*n* = 311, 67.8%), while 120 (26.1%) reported 1–2 previous concussions, and 28 (6.1%) reported 3 or more concussions. Of participants enrolled in a sport preparatory program, the majority participated in team sports (72.0%), with soccer (32.1%) and handball (30.9%) being most common. Sociodemographic variables stratified by concussion group are presented in Table 1.

**Table 1:** Participant Demographics by Concussion History Group

	Concussion History							
Variable	0 (*n* = 311)		1–2 (*n* = 120)		3+ (*n* = 28)		*χ* ^*2*^	*p*_*adj*_
Age, *M* ± *SD*	16.8 ± 0.8		16.8 ± 0.9		16.9 ± 0.9		0.128^*H*^	0.99
BMI, *M* ± *SD*	21.9 ± 2.7		22.5 ± 3.2		22.1 ± 2.7		4.097^*H*^	0.34
Sex (male), *n* (%)	181 (58.2%)		70 (58.3%)		16 (57.1%)		0.015	0.99
Year 1, *n* (%)	147 (47.3%)		58 (48.3%)		14 (50.0%)		1.394	0.99
Year 2, *n* (%)	109 (35.0%)		46 (38.3%)		10 (35.7%)			
Year 3, *n* (%)	55 (17.7%)		16 (13.3%)		4 (14.3%)			
Sports-tailored program, *n* (%)	157 (50.5%)		71 (59.2%)		14 (50.0%)		2.709	0.55
Team sport, *n* (%)	107 (68.2%)		59 (81.9%)		9 (64.3%)		8.451	0.05
Live at home with parents, *n* (%)	299 (96.1%)		112 (93.3%)		26 (92.8%)		1.857	0.66
ADHD/ASD/LD, *n* (%)	25 (8.0%)_a_		20 (16.7%)_b_		7 (25.0%)_b_		11.965	0.038
Lifetime gambling, *n* (%)	144 (46.3%)		59 (49.2%)		14 (50.0%)		0.374	0.99
Past 30 days gambling, *n* (%)	78 (25.1%)		28 (23.3%)		7 (25.0%)		0.145	0.99
Lifetime parental gambling, *n* (%)	108 (34.7%)		40 (33.3%)		10 (35.7%)		0.096	0.99

Note. *N* = 459. ^*H*^Kruskal-Wallis chi-squared test statistic. *χ*^*2*^ = Chi-square test statistic. BMI = body mass index. ADHD = attention deficit hyperactivity disorder. LD = learning disorder. Test *p*-values were adjusted to account for multiple testing using the Benjamini and Hochberg (52) procedure. Proportions denoted with the same letter subscript do not statistically differ based on *post hoc* Chi-square tests

Chi-square analyses indicated that there were differences in the percentages of participants diagnosed with ADHD, ASD, or LD across concussion groups *χ*^*2*^(2) = 11.965, *p* = .003, *p*_*adj*_ = 0.038, *V* = 0.161. Post hoc pairwise comparisons revealed that fewer participants with no concussion history reported a diagnosis than those with three or more concussions, *χ*^*2*^(1) = 8.645, *p* = .003, *p*_*adj*_ = 0.025, *V* = 0.160, and those with 1–2 concussions, *χ*^*2*^(1) = 6.894, *p* = .009, *p*_*adj*_ = 0.043, *V* = 0.126. The percentage of participants with a diagnosis did not differ between participants with 1–2 concussions and those with three or more, *χ*^*2*^(1) = 1.057, *p* = .30, *p*_*adj*_ = 0.57, *V* = 0.085.

### Effect of concussion history on psychological distress and hazardous gambling

**Fig. 1:**
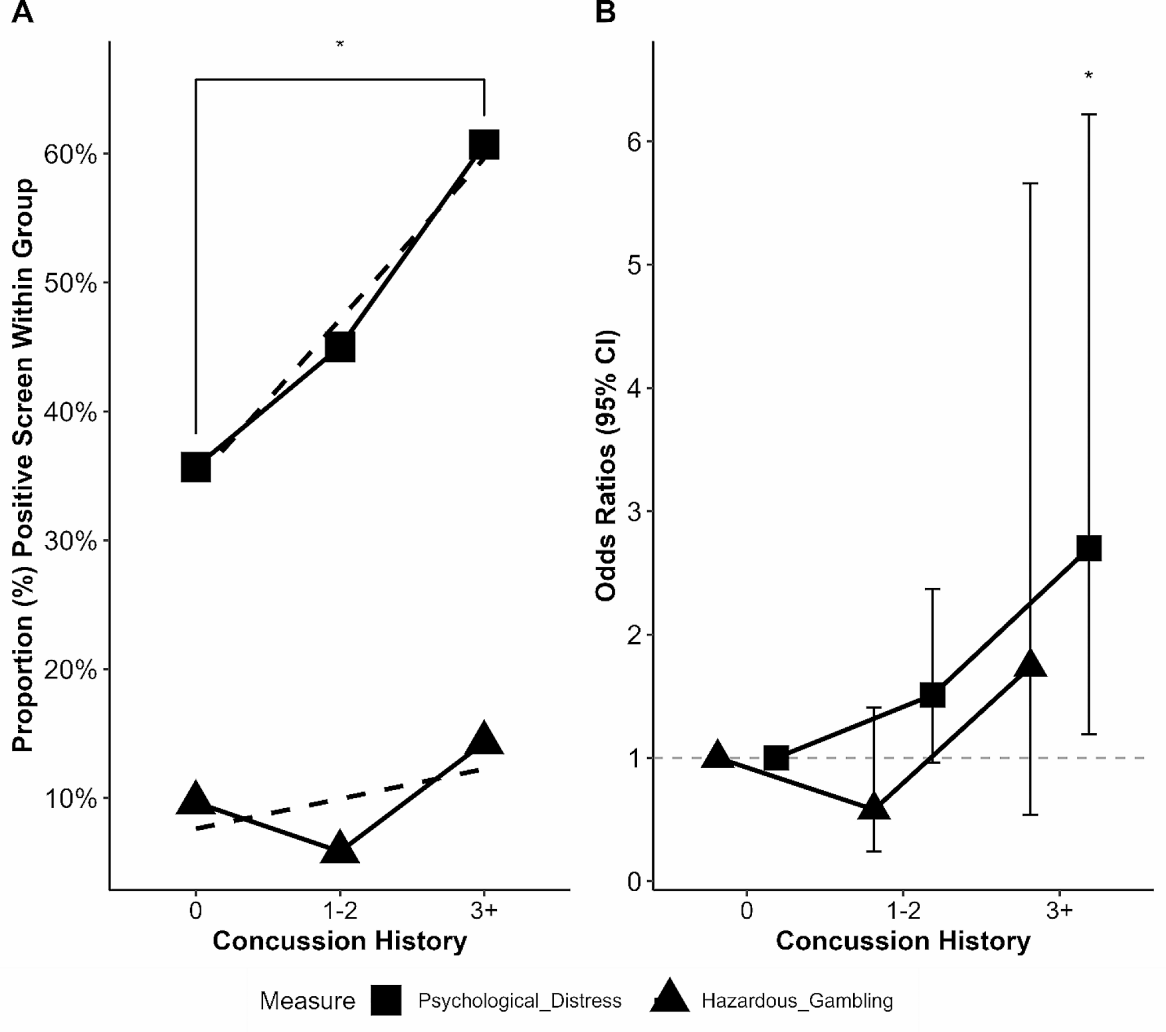
Moderate-Severe Psychological Distress and Hazardous Gambling Across Concussion Group. *Note*. Concussion history as a predictor of psychological distress (K6) and hazardous gambling (NODS-CLiP). **A**: Crude differences in proportions between concussion groups screening positive for moderate to severe psychological distress and hazardous gambling. The proportion of participants with 3 + more concussions reporting moderate to severe psychological distress was significantly higher than those with no concussion history. No differences were found in hazardous gambling across concussion groups. Dotted lines illustrate linear fitted model. **B**: Adjusted odds ratios with 95% confidence intervals (CI) for logistic regression models predicting outcome variables while controlling for covariates (Model 2a & 2b). Firth regression used for hazardous gambling model. The odds ratio predicting moderate to severe psychological distress from concussion history was significant for those with 3 + concussions. Psychological Distress = positive K6 screen. Hazardous Gambling = positive NODS-CLiP screen. *p < .05

Binomial logistic regressions were performed to ascertain the effect of concussion history on the likelihood that participants were to report moderate to severe psychological distress. In the crude model only including concussion history as a predictor (Model 1a), only those sustaining three or more concussions were more likely to exhibit moderate to severe psychological distress, having 2.78 times greater odds than those with no concussion history. In the adjusted model including age, sex, athlete status, and psychiatric history as covariates (Model 1b), sustaining three or more concussions remained a significant predictor of moderate to severe psychological distress, having 2.71 higher odds than those with no concussion history. Additional binomial logistic regressions were performed to assess the crude effect of concussion history on the likelihood that participants were to exhibit hazardous gambling. In both the crude model only including concussion history as a predictor (Model 1b) and the adjusted model including age, sex, athlete status, psychiatric history, and lifetime parental gambling as covariates (Model 2b), concussion history was not predictive of hazardous gambling. Model parameters are presented in Table 2. Crude models of the effect of concussion history on the percentage of participants screening positively for moderate to severe psychological distress and hazardous gambling are illustrated in Fig. 1A, while odds ratios for adjusted models including covariates predicting positive screenings (Model 2a & 2b) are illustrated in Fig. 1B.

**Table 2:** Logistic Regression Models Predicting Psychological Distress and Hazardous Gambling

Variable	B	SE	*p*	OR [95% CI]
Psychological Distress				
Model 1a				
Intercept	-0.59	0.12	< 0.001	
1–2 Concussion Hx (0)	0.39	0.22	0.08	1.47 [0.96, 2.26]
**3 + Concussion Hx (0)**	**1.02**	**0.40**	**0.011**	**2.78 [1.26, 6.16]**
Model 2a				
Intercept	-2.37	2.01	0.24	
Age	0.08	0.12	0.49	1.08 [0.86, 1.37]
Sex (Female)	0.98	0.20	< 0.001	2.66 [1.78, 3.97]
Athlete status (Athlete)	-0.15	0.20	0.45	0.86 [0.57, 1.28]
ADHD/ASD/LD	0.52	0.31	0.10	1.68 [0.91, 3.10]
1–2 Concussion Hx	0.39	0.23	0.09	1.47 [0.94, 2.31]
**3 + Concussion Hx**	**1.00**	**0.42**	**0.018**	**2.71 [1.19, 6.18]**
Hazardous Gambling^*a*^				
Model 1b				
Intercept	-2.25	0.19	< 0.001	
1–2 Concussion Hx	-0.49	0.43	0.22	0.61 [0.26, 1.42]
3 + Concussion Hx	0.53	0.56	0.36	1.70 [0.56, 5.10]
Model 2b				
Intercept	-13.94	3.50	< 0.001	
Age	0.71	0.20	< 0.001	2.04 [1.37, 3.04]
Sex (Female)	-1.78	0.49	< 0.001	0.16 [0.06, 0.44]
Athlete status (Athlete)	-0.45	0.35	0.19	0.64 [0.32, 1.27]
ADHD/ASD/LD	-0.05	0.57	0.93	0.95 [0.31, 2.91]
Parental gambling	0.62	0.35	0.07	1.85 [0.93, 3.70]
1–2 Concussion Hx	-0.55	0.45	0.20	0.58 [0.24, 1.40]
3 + Concussion Hx	0.56	0.59	0.35	1.76 [0.55, 5.63]

Note. *N* = 459. ADHD = attention deficit hyperactivity disorder. LD = learning disorder. Hx = history. Reference for binary and ordinal variables included in parentheses. Significant results relevant to study hypotheses are bolded. ^a^Logistic regression using Firth method with post hoc intercept corrections

## Discussion

Concussion and TBI have been linked to the development of long-term mental health issues and addictive behaviors [5–9, 23, 25–27], yet little research has targeted psychological distress in adolescent students nor examined the relationship between concussion and adolescent hazardous gambling. Accordingly, the present study evaluated how concussion history was associated with psychological distress and hazardous gambling while controlling for important individual differences. Assessing this relationship is essential for (a) accurately informing individuals of the risks involved in activities that expose them to biomechanical impacts (sports, military service, etc.), (b) informing clinicians of potential sequelae of concussive injuries to better meet their patients’ needs, and (c) providing a better picture for how we can proactively reduce the risk and severity of concussion.

Controlling for important covariates associated with concussion, psychological distress, and hazardous gambling, we found that concussion history was associated with increased psychological distress for students reporting three or more previous concussions, but not associated of hazardous gambling, which partially supports our hypotheses (Fig. 1). According to Models 1a and 2a, only in students that self-reported three or more concussions was there an increase in odds of moderate to severe psychological distress. These findings are in line with a growing body of literature highlighting that concussions can lead to increases in psychological distress over time [5, 9]. Non-significant results regarding associations between concussion and hazardous gambling contrasts studies indicating that TBI or concussion is associated with hazardous gambling [23–27]. Including covariates proved to be essential in more accurately assessing the effects of concussion history on outcomes. As expected, age and gender were important confounders to account for. Being 18 years or older was associated with greater odds of endorsing hazardous gambling, while identifying as female was associated with greater psychological distress but lower odds of hazardous gambling, which is consistent with similar studies in adolescent populations [19, 31, 41].

There are several possible explanations as to why only students with three or more concussions were more likely to endorse moderate to severe levels of psychological distress. Sustaining multiple concussions can be psychologically traumatic and have a lasting negative impact on an individual. For example, the increased likelihood of sustaining an additional concussion after the initial injury [40, 57] can contribute to the fear and anxiety an individual experiences returning to the modality where the injury occurred (e.g., sport, military duty, driving) [58, 59]. In addition, being forcibly sidelined and subjected to recovery procedures multiple times can be stressful and depressing for individuals that rely heavily on, for example, sports for social connection, physical conditioning, structure, and overall well-being [58]. Similarly, there are several possible explanations as to why hazardous gambling was not associated with concussion history. Primarily, gambling is a restricted activity for people under the age of eighteen in Sweden [60], and therefore less accessible and available to a large proportion of the students who participated in the present study. Secondly, despite evidence from preclinical research suggesting potential mechanisms for a causal link between brain injury and hazardous gambling [28, 29], no brain imaging studies in the clinical context testing this theory exist currently. Likewise, whether gambling is a common strategy to cope with concussion-induced chronic symptoms has not been explored. Lastly, previous studies demonstrating a relationship largely recruited participants who had sustained moderate-to-severe TBI injuries or recruited participants who were older [23–27]. It may be that gambling behavior is not affected by TBIs of lower severity. Future research detailing how neurobiological changes and psychological responses to concussion contribute to changes in gambling behavior may aid in elucidating this relationship and yield more conclusive results.

### Limitations

The current study’s findings must be interpreted carefully considering its limitations. Most importantly, causation cannot be assessed due to the cross-sectional design of the study. The onset of psychological distress and hazardous gambling behavior may have occurred prior to concussion, or the time required for symptoms to manifest after concussion was insufficient. Concerning our recruitment strategy, we cannot report an accurate overall response rate from students, as it behooved principals to distribute the survey link to instructors. Despite more than half of the schools agreeing to participate, the exclusion of students from both non-participating schools and, likely, a portion of students from participating schools who were not invited for unknown reasons introduces a degree of sampling bias. In relation to the measures used in the present study, the NODS-CLiP questionnaire used is a brief form that has the potential of being overly sensitive, meaning that recreational, non-problematic gamblers may screen positively, possibly leading to spurious results [47]. In addition, our assessment of concussion history did not evaluate the severity of previous concussive injuries, which may have contributed to greater variation in the relative burden of injury sustained across concussions. Similarly, concussion history as well as other measures were self-reported and subject to bias. However, self-reported concussion history was preferred in the present study to avoid the underreporting associated with requiring that concussions had to have been diagnosed by a medical professional [61]. Furthermore, the present study was unable to account for all known comorbid and pre-morbid covariates (history of depression, alcohol use, sleep disturbance) associated with the development of hazardous gambling and psychological distress. Lastly, the sample size may have not yielded sufficient statistical power to assess these relationships accurately considering the inherent heterogeneity of concussive injuries. Future research would benefit from having larger sample sizes which would enable far more comprehensive models to assess the relationships between concussion, gambling behavior, and psychological distress.

## Conclusions

In sum, our findings imply that mental health evaluations beyond the acute phase of injury in adolescents and young adults who have sustained multiple concussions may be of relevance to clinicians in identifying and managing post-acute concussion sequelae. Additionally, these preliminary data do not provide support probing for hazardous gambling in adolescents who have sustained multiple mild brain injuries. The nature of this connection will be better understood by subsequent research conducted with larger samples.

## Data Availability

Data can be made available upon request.
